# Investigation of the relationship between neuroplasticity and grapheme-color synesthesia

**DOI:** 10.3389/fnins.2024.1434309

**Published:** 2024-08-19

**Authors:** Nadine Eckardt, Christopher Sinke, Stefan Bleich, Ralf Lichtinghagen, Markus Zedler

**Affiliations:** ^1^Department for Psychiatry, Social Psychiatry and Psychotherapy, Hannover Medical School, Hanover, Germany; ^2^Department of Psychiatry, Social Psychiatry and Psychotherapy, Division of Clinical Psychology & Sexual Medicine, Hannover Medical School, Hanover, Germany; ^3^Institute of Clinical Chemistry, Hannover Medical School, Hanover, Germany

**Keywords:** synesthesia, neuroplasticity, brain-derived neurotrophic factor, connectivity, hyperconnected brain, embryonic relict, development, cognition

## Abstract

Grapheme-color synesthesia is a normal and healthy variation of human perception. It is characterized by the association of letters or numbers with color perceptions. The etiology of synesthesia is not yet fully understood. Theories include hyperconnectivity in the brain, cross-activation of adjacent or functionally proximate sensory areas of the brain, or various models of lack of inhibitory function in the brain. The growth factor brain-derived neurotrophic (BDNF) plays an important role in the development of neurons, neuronal pathways, and synapses, as well as in the protection of existing neurons in both the central and peripheral nervous systems. ELISA methods were used to compare BDNF serum concentrations between healthy test subjects with and without grapheme-color synesthesia to establish a connection between concentration and the occurrence of synesthesia. The results showed that grapheme-color synesthetes had an increased BDNF serum level compared to the matched control group. Increased levels of BDNF can enhance the brain's ability to adapt to changing environmental conditions, injuries, or experiences, resulting in positive effects. It is discussed whether the integration of sensory information is associated with or results from increased neuroplasticity. The parallels between neurodegeneration and brain regeneration lead to the conclusion that synesthesia, in the sense of an advanced state of consciousness, is in some cases a more differentiated development of the brain rather than a relic of early childhood.

## 1 Introduction

Synesthesia is a norm variant of human perception in which two or more different sensory perceptions are linked together. For example, visual observation of a word may lead to a visual perception of color or taste in another sensory modality (Ward et al., 2005). In 2001 Grossenbacher and Lovelace introduced the terms “inducer” for the triggering stimulus, as well as the directly associated additional perceptual event, the so-called “concurrent” (Grossenbacher and Lovelace, 2001). Over 80 different types of synesthesia have been identified so far (Day, 2016) and recent research indicates that this list will likely continue to grow (Cytowic and Eagleman, 2011).

The “inducer-concurrent paring” is idiosyncratic, individually unique and non-random, so that a specific accompanying sensation can be attributed to each triggering stimulus, which occurs automatically, inevitably and independently of the synesthete's will (Mills, 1999; Mattingley et al., 2001; Lupiáñez and Callejas, 2006). Usually, it is an unidirectional perception (Grossenbacher and Lovelace, 2001), but in principle it can also occur indirectly subconsciously in both directions (Brugger et al., 2004; Cohen Kadosh et al., 2007; Meier and Rothen, 2007). Synesthetic perception is consistent and reproducible throughout life (Baron-Cohen et al., 1987; Cytowic, 2002; Simner and Logie, 2008). Although, some studies suggest that this perception may vary over time (Meier et al., 2014; Simner et al., 2017; Chromý et al., 2019). Another possible classification is described by Simner (2012) and does not focus on the sensory quality and consistency but rather characterizes synesthesia according to the neurobiological underlying mechanisms as to capture all forms of synesthesia (Simner, 2012).

This norm variant of human consciousness occurs in over 4% of the population (Simner et al., 2006). With a prevalence of about 1–2%, grapheme-color synesthesia is a common type of synesthesia (Simner et al., 2006; Carmichael et al., 2015; Simner and Carmichael, 2015), and has thus been of great research interest.

Grapheme-color synesthesia is characterized by the perception of color, triggered by graphemes such as numbers or letters. The perceived synesthetic sensation can either be projected into the “real external world” or is only visible in front of the “inner monitor/eye.” The former are called “projectors,” the latter “associators” (Smilek et al., 2001; Dixon et al., 2004).

While the underlying foundations of hyperconnectivity in synesthesia are still to be fully elucidated, several explanatory models for the etiology of synesthesia have been proposed. While two underlying mechanisms have been proposed, the models are not mutually exclusive, as both suggest that hyperconnectivity in the brain cause synesthesia. The hyperconnectivity may be an embryonic relict, not regressing in the brain of newborns (Maurer and Maurer, 1988; Maurer and Mondloch, 2005) or it could arise from more developed brain structures, leading to the development of a heightened form of consciousness. At the neurophysiological level, there is ongoing discussion regarding the structural and functional connectivity of the brain, especially concerning the development of the aforementioned hyperconnectivity. Essentially, two variants are proposed: (a) cross-activations due to direct connections of functionally or structurally neighboring cerebral sensory areas (Ramachandran and Hubbard, 2001; Hubbard et al., 2011), and (b) missing or reduced inhibitory function within the brain, e.g., in the form of the disinhibited-feedback model (Grossenbacher and Lovelace, 2001; Neufeld et al., 2012) or the re-entrant-feedback model (Smilek et al., 2001).

Synesthetes are an ideal group to study altered communication in the brain. Their condition is not influenced by additional factors often associated with alterations in brain connectivity, as can be observed in other diseases or disorders, such as e.g., autism (Hull et al., 2017; Haghighat et al., 2021). Thus, they are more straightforward to phenotype and provide insights into hyper- or hypo-connectivity and mechanisms of cross-modal sensory processing in these syndromes (Tomson et al., 2011).

To gain a deeper understanding of the neurobiological and etiological underpinnings of synesthetic perception, numerous neuroimaging and neurophysiological studies have been conducted. These studies have provided valuable insights into the mechanisms underlying synesthesia. Several studies using functional magnetic resonance imaging (fMRI) have shown increased brain activity compared to non-synesthetes during viewing or auditory perception of graphemes in traditional color processing areas, from the primary visual cortex corresponding to area V1 to visual association areas, especially activation of color area V4/V8 (Nunn et al., 2002; Hubbard et al., 2005). Conversely, some studies did not report any activation in the color-processing region V4 (Hupé et al., 2012; Sinke et al., 2012). However, other brain areas, such as the parietal cortex, insula, operculum, precentral gyrus and prefrontal cortex also appear to have a functional role. So far, no single pattern has emerged, but rather a cooperation of different areas seems to be involved (Rouw et al., 2011).

On the neurophysiological level, Schlitz (1999) discovered distinct P300 components of event-related potentials (ERP) using EEG signals, especially in the prefrontal and frontal areas, which was interpreted as inhibition of neuronal activity in these areas (Schlitz, 1999).

Understanding structural brain differences between synesthetes and non-synesthetes is focus of ongoing research and discussions. Currently, main investigation methods include voxel-based morphometry (VBM), which identifies local increases in the tissue volume and density of gray matter, and diffusion tensor imaging (DTI), which detects enhanced structural connectivity, with greater white matter coherence, by employing differences in microstructure along with tractography or nerve fiber integrity. For instance, Rouw and Scholte observed disparities between individuals with synesthesia and those without, in terms for both gray (Rouw and Scholte, 2010) and white matter (Rouw and Scholte, 2007), respectively. However, more recent studies have produced contrasting results regarding the structural differences. For instance, employing VBM, DTI, and sulcus-based morphometry methods with the “New Statistics” approach, Dojat et al. (2018) found no structural cerebral differences in 10 or 22 grapheme-color synesthetes (in the 2nd part of the study) compared to 25 control subjects. In contrast, Arend et al. (2018) showed increased gray matter in 19 synesthetes compared to controls in the left cerebellum, as well as in parts of the limbic system, namely the right amygdala. However, the trigger itself appears to impact synesthetic perception, as indicated by an increase in gray matter within the left angular gyrus, a higher associated area, only found in synesthetes who reported numbers as the stimulus for triggering, compared to other synesthetes (Arend et al., 2018).

In summary, various imaging techniques indicate increased brain activity and altered brain structure between synesthetes and non-synesthetes. However, they do not provide a consistent picture, specific brain region(s) or a shared mechanism of communication between distinct brain areas.

Furthermore, the discussed neuroimaging results should be interpreted with caution, as Hupé and Dojat (2015) expressly pointed out in a review. The used methodological and statistical approach as well as the sample size should be checked carefully to ensure the validity of subsequent conclusions. It was proposed that, synesthesia should be regarded as a kind of “childhood memory” that cannot (yet) be detected by the most recent technique (Hupé and Dojat, 2015).

As demonstrated above, the origin of enhanced sensory connectivity in synesthetes remains uncertain. Nonetheless, compared to non-synesthetes, the reported structural and functional differences give rise to the theory that synesthetic perceptions may be associated with increased connectivity between concurrent and inducer processing areas.

At the genetic level, there seems to be a hereditary predisposition, but so far no uniform pattern of inheritance could be identified. Instead, there is locus heterogeneity (Asher et al., 2009; Tomson et al., 2011). A recent genetics study by Tilot et al. (2018), has revealed a new aspect of nervous system morphogenesis. Investigation of three families with cross-generational sound-color synesthesia, leading to the detection of six genes associated with cell integration and axon genesis. Expression of these genes occurs in both the infant and adult brain (Tilot et al., 2018).

Neuroplasticity, the experience-dependent structural, functional, and adaptive capacity of the brain to change, can occur through life; the underlying plastic processes of adaptation manifest themselves at multiple levels, both in the developing and in the adult brain (Gulyaeva, 2017). Neurotrophins, as neuromodulators, play a crucial role in this process. Along with nerve growth factor (NGF), neurotrophin 3 (NT3), and neurotrophin 4 (NT4), the brain-derived neurotrophic factor (BDNF) is a key part of this family (Kowiański et al., 2018). The growth factor BDNF has been one of the most studied neurotrophic factors since its discovery by Barde in 1982 (Barde et al., 1982; Binder and Scharfman, 2004). BDNF is present in both the central and peripheral nervous systems, as well as in peripheral organs, platelets, as well as smooth muscle, endothelial, and activated inflammatory cells.

BDNF is able to cross the blood-brain barrier (Pan et al., 1998). Indeed, several studies in animal models have shown that BDNF serum concentration may correlate with levels in the central nervous system, however, this remains disputed (Sartorius et al., 2009; Elfving et al., 2010a; Klein et al., 2011). In a direct comparison between brain and serum BDNF, Karege et al. found similar BDNF changes during maturation and aging in rat brains compared to serum levels (Karege et al., 2002b). Therefore, it seems that circulating BDNF levels correspond to the central concentration.

BDNF is active in two signaling mechanisms, binding with both: the tyrosine kinase receptor-B (Trk-B) and the p75 neurotrophin receptor (p75NTR) (Sandhya et al., 2013). Through these complex signaling pathways BDNF influences a wide range of functions including morphological development, various neuronal functions and neuroplasticity of the brain. BDNF is relevant for both progressive and regressive processes. BDNF plays a central mediating role in a competitive neuronal environment, as the degeneration of inactive neuronal cells directly depends on the concentration of BDNF (Cao et al., 2007). Singh et al. (2008) investigated the mechanism behind this neuronal pruning, in more detail, specifically when activity-based release of BDNF activates p75NTR in neighboring, inactive neuronal cells. This triggers a signaling cascade, leading, mainly, to inhibition of the protective TrkA signaling pathway and ultimately degeneration of the cells. It therefore seems that active axons possess the capability to eliminate unstimulated, competing axons. In this competition, BDNF fulfills two distinct functions: firstly, it protects active neuronal cells, and secondly, it facilitates the degeneration of redundant or no longer functional neuronal connections. The activity-dependent pruning of neuronal circuits and the refinement of connections through competition ultimately lead to specification and optimization of these networks. In the developing brain, the role of BDNF, furthermore, includes neurogenesis, gliogenesis, and the dynamic assembly and disassembly of axon branching and dendritic spinous processes. In addition to neuronal differentiation, proliferation and migration, BDNF also has a modulatory role in pre- and postsynaptic differentiation in terms of synaptic connectivity of neurons (Huang and Reichardt, 2001; Cohen-Cory et al., 2010). In the adult brain, it further plays a crucial role in in neuroprotection or maintenance of existing neurons (Hu et al., 2005). It also increases brain activity, leading to enhanced neuroplasticity, which is crucial for improving cognitive processes such as memory and cognition abilities (Cunha et al., 2010; Gonzalez et al., 2016; Sasi et al., 2017; Kowiański et al., 2018).

This wide range of effects makes BDNF the ideal candidate for monitoring changes in neuronal structure and function.

Peripheral BDNF is stored in platelets at a rate of more than 90% and is released in an activity-dependent manner, allowing for measuring with an Enzyme-Linked Immunosorbent Assay (ELISA) method (Radka et al., 1996; Fujimura et al., 2002).

The detection of circulating BDNF levels has become an area of great interest in recent years due to their association with various neuropsychiatric disorders such as Parkinson's disease (Jiang et al., 2019), autism spectrum disorder (Liu et al., 2021), depression (Shi et al., 2020), and Alzheimer's disease (Ng et al., 2019). Objective evaluations of these levels have been a focus in current research.

The study aims to gain a better understanding of the proposed hyperconnectivity in synesthetes. As BDNF is a marker of plasticity and plasticity the foundation of building new connections in the brain, it is hypothesized that synesthetes have a higher BDNF levels as a neuronal prerequisite for a hyperconnected brain. To ascertain these increased serum BDNF concentrations, blood samples were analyzed from both.

## 2 Materials and methods

### 2.1 Subjects

**Figure 1:**
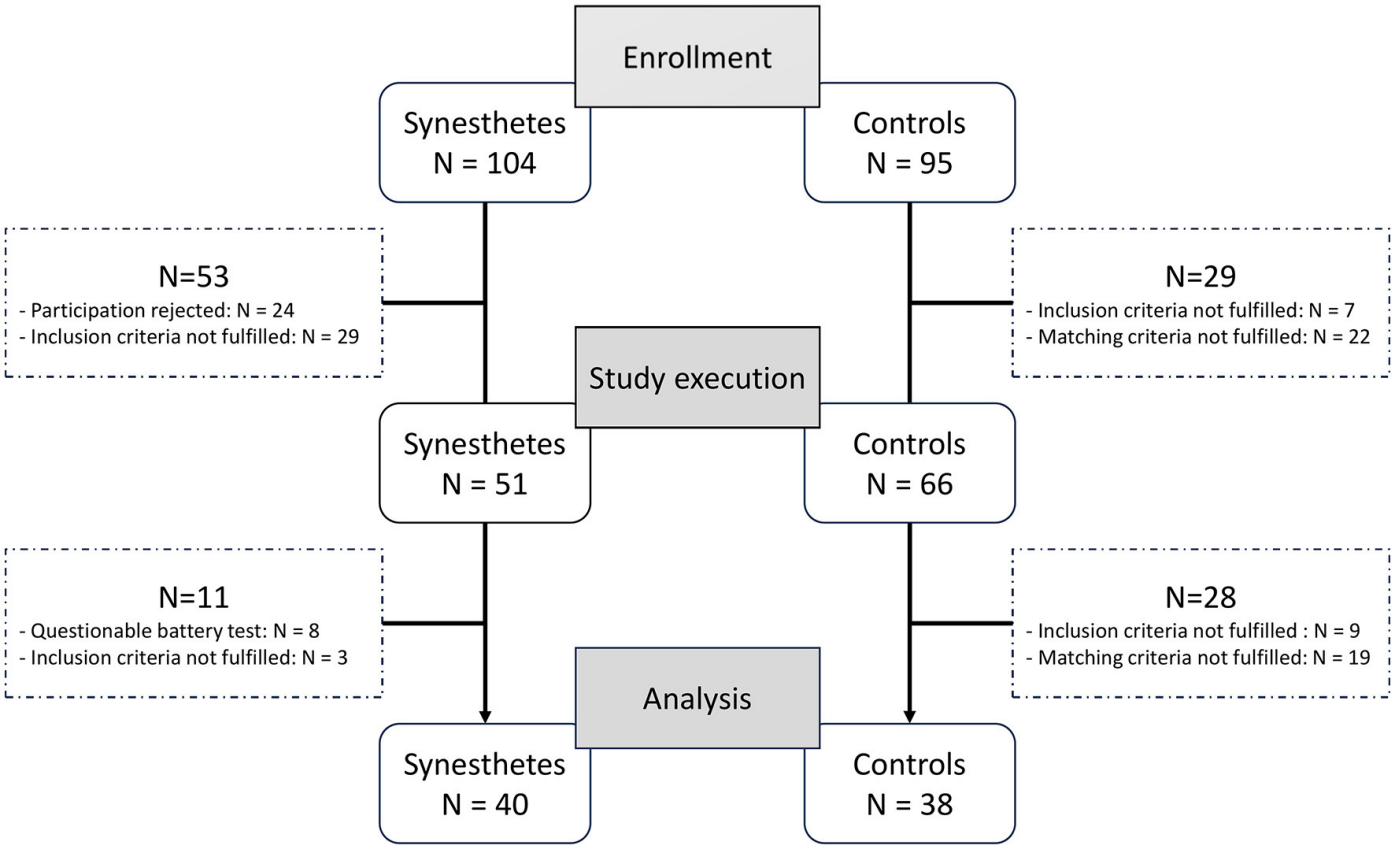
Consort flowchart about the study design. Gray rectangles represent the study stages, round corner rectangles indicate the number of participants in each stage, and dotted rectangles indicate the number of subjects excluded from the study due to various criteria.

The investigated group of synesthetes is composed of a pre-existing collective who consented to participate, in addition to volunteers who were recruited after the study's announcement through the “German Synesthesia Society.” The control group was mainly recruited in the Hannover Medical School and the University of Veterinary Medicine Hannover. The consort flowchart about the study is seen in Figure 1 summarizing the losses due to exclusion criteria.

A total of 104 potential synesthetes and 95 control subjects were registered for the study.

To limit the number of possible influencing factors, the following exclusion criteria were used: history of traumatic neurological diseases or existing neurological diseases, mental illnesses, inflammatory diseases, especially autoimmune diseases, disorders of the cardiovascular system, kidney function or hematopoiesis, diabetes mellitus, vitamin deficiency diseases, substance dependence or abuse, pregnancy, and lactation. Only healthy female subjects aged 18–75 years were included in the study.

Using the aforementioned criteria and excluding non-responsive subjects, we selected participants matched for age. This resulted in 117 individuals (51 with synesthesia and 66 controls) being included in the study.

The self-reported group affiliations of those subjects were validated using David Eagleman's synesthesia battery test. Based on the results the participants were divided into “synesthetes” (score ≤ 1) and “controls” (score > 1). A more detailed description of the test is given in Section 2.3.

All non-synesthetes achieved a value of >1 and none was excluded from the study on the grounds of low constancy scores. In contrast, some self-reported synesthetes exhibited scores >1, which rendered their synesthesia classification more challenging. Consequently, they were excluded from the study.

However, scores of the test were not used as exclusion criteria alone. Instead, the subjects were further pretested with the aid of an anamnesis questionnaire, a medical interview, and a physical examination consisting of an internal and neurological check-up. From these investigations confounding and accompanying variables that could affect sensory perception or BDNF serum levels were identified to avoid bias of the study outcome.

Apart from the test score, all exclusion criteria were identical for both groups. In total *N* = 4 synesthetes were excluded solely due to the score value, *N* = 2 did not perform the battery test, *N* = 2 had a higher score but also failed other inclusion criteria, and lastly *N* = 3 failed the pretests. For the controls *N* = 9 failed the pretests and *N* = 19 were excluded in order to match the two groups, leaving a total of 40 healthy synesthetes and 38 subjects in the control group. For these 78 subjects serum BDNF levels were investigated.

### 2.2 Clinical characterization

In addition to a primary classification according to age and sex, subjects were differentiated according to educational level and general lifestyle habits such as current smoking (Yes/No), regular consumption of alcohol and drugs (Yes/No), and sports activities (Yes: ≥1 per week sports, No: regular walking or no sports). In addition, neurological-physiological and internal diseases were also determined (Yes/No).

Ultimately, 40 synesthetes and 38 non-synesthetes could be included in the study.

### 2.3 Verification of synesthetic perception

The standardized and validated “The Synesthesia Battery Test” by Eagleman et al. (2007), which is freely available online,^1^ was used to accurately record and characterize synesthesia (Carmichael et al., 2015). During the three trial runs of the “Grapheme-color mapping test,” synesthetes exhibited significantly greater consistency (score ≤ 1.0), compared to non-synesthetes, with a significantly higher score (usually ~ 2.0).

The subsequent “Speed-Congruency-Test” “verifies” the synesthetic color perception and rules out memory strategies as a factor, since synesthetes intuitively perceive their colors and do not rely on memory strategies, which usually takes time and is also prone to errors. However, here the speed test is not used as hard exclusion criteria as e.g., some participants with a score between ~75 and 85% are still included in the study (*N* = 6). They had a low consistency score but demonstrated difficulty in utilizing the computer and a perceived sense of time pressure to respond promptly. This resulted in some participants inadvertently clicking the wrong button.

Furthermore, after completing the test, synesthetes were asked to report further forms of synesthesia they experience from a list of common types. In this evaluation, the weekday-color-synesthesia and month-color-synesthesia were included in the grapheme-color-synesthesia category. Pitch-color, chord-color and musical instrument-color synesthesia were also combined as music-color synesthesia.

### 2.4 Determination of the BDNF concentration

To avoid methodological errors and thus inter-sample variants, the same procedure was followed with all samples. Furthermore, sample collection and storage were performed strictly according to the instructions of the BDNF data sheet (Aviscera Bioscience Inc, 2017).

Peripheral venous blood was collected using a SARSTEDT serum monovette (S-Monovette^®^ 5.5 ml, serum with clot activator). After 30 min of clotting time, in an upright position, the serum monovettes were centrifuged at 1,000 *g* for 15 min at 20°C. A SARSTEDT Seraplas^®^ V15 valve filter was subsequently used to ensure optimal separation of serum from the blood cells. The serum was pipetted as an aliquot in 2 ml Eppendorf tubes from SARSTEDT and frozen at −80°C to ensure sufficient stability of BDNF over the storage period of ≤ 15 months (Bus et al., 2011). Subsequently, the BDNF serum concentration of all samples were measured together and blinded for “Synesthesia” and “Controls” with two ELISA assays from the same batch. For this, the sandwich “Brain-derived neurotrophic factor (BDNF; Human, Mouse, Rat) ELISA KITs” from AVISCERA BIOSCIENCE was used. The procedure was strictly in accordance with the manufacturer's specifications. The samples were diluted 40-fold and measured in duplicate at a wavelength of 450 nm, as were the comparison samples (zero sample, standard curve that ranged from 234 to 1,500 pg/ml BDNF and positive control). The mean of these results was used for statistical analysis.

### 2.5 Statistical analysis

The differences between synesthetes and non-synesthetes based on the primary classifications (comorbidities, smoking, sports activities, sleep and stress behavior, BDNF serum level, etc.), were investigated. For categorical variables (e.g., cardiovascular disease, smoking), group differences were examined using Fischer's exact test, whereas for metric scale level (e.g., age, BDNF serum level), a two sided *t*-test between groups was performed after testing for normal distribution using the Kolmogorov-Smirnov test. If there was no normal distribution, a non-parametric test was used.

The second approach focuses on identifying possible influencing factors in relation to the BDNF serum level. Initially, we univariately determined which factors might have a relationship with BDNF serum level. For this purpose, after prior testing for normal distribution, a two-tailed *t*-test was performed for dichotomous variables and an ANOVA analysis for categorical influencing factors.

Subsequently, a multivariate linear regression model with backward selection was used to evaluate the influence of synesthesia on BDNF levels after elimination of possible confounding factors. Therefore, all influencing factors that had a univariate significant effect on the target variable (here, a *p*-value < 0.2 was chosen) were included in the multivariate model. Afterwards, a backward selection to the 5% significance level was performed to identify variables that were statistically significantly associated with the target variable.

For all analyses, *p* < 0.05 was assumed to be significant. If a normal distribution was not given or the sample size N was small, additional non-parametric tests were performed. If both test procedures yield the same result, the parametric test is reported, as this facilitates the calculation sample size for a possible follow-up study. The exploratory nature of this study should also be noted.

SPSS, version 2023 was used for all analyses.

## 3 Results

### 3.1 Clinical characteristics of the subjects

In this pilot study, 40 grapheme-color synesthetes aged between 18 and 69 years and 38 age-matched control subjects ranging in age from 19 to 70 years were evaluated for their serum BDNF levels (Synesthetes: M = 40.85 ± 15.27 years; control group: M = 39.87 ± 14.88 years, *t*_(76)_ = 0.287, *p* = 0.775).

No significant difference could be found regarding educational level [university: synesthetes *N* = 17, controls *N* = 15, high school: synesthetes *N* = 16, controls *N* = 14, secondary school: synesthetes *N* = 7, controls *N* = 9, $\chi_{\left(1,\text{N}=78\right)}^{2}$ = 0.457, *p* = 0.796].

Similarly, no significant difference was found with respect to BMI [Synesthetes: M = 24.07 ± 4.3; control group: M = 23.6 ± 3.6, *t*_(76)_ = −0.486, *p* = 0.628].

Furthermore, no group differences were found in the other potential influencing factors, as summarized in the Table 1.

**Table 1:** Possible influencing factors elicited by anamnesis and physical examination in absolute numbers, as well as their Fischer and *p*-values.

	**Synesthetes**		**Control group**		
**Disease**	**Yes**	**No**	**Yes**	**No**	***r*** **/** ***p*** **-value**
Recovered neurological-psychological disease	7^*^	33	2^*^	36	0.155
Cardiovascular system	2	38	4	34	0.425
Asthma	2	38	0	38	0.494
Thyroid disease	4	36	6	32	0.512
Allergies	16	24	11	27	0.348
Autoimmune disease	3	37	0	38	0.241
Smoking, current	4	36	1	37	0.359
Py	*M* = 7.25—*SD* = 6.46		*M* = 5.42—*SD* = 5.55		
Sport	27	13	29	9	0.455

### 3.2 The synesthesia battery test

Significant differences could be determined between synesthetes and control group with respect to the color congruency “grapheme-color test” [*t*_(76)_ = −22.345, *p* ≤ 0.01] and accuracy “speed-congruency test” [*t*_(76)_ = 19.455, *p* ≤ 0.01]. In the latter test, both groups showed a similar reaction time [*t*_(76)_ = −0.123, *p* = 0.903]. Mean values and standard derivations are summarized in Table 2.

**Table 2:** Results of the synesthesia battery test—mean values, standard derivations and *p*-values.

	**Synesthetes**	**Control group**	***p*-value**
	***N*** = **40**	***N*** = **38**	
Grapheme color score	*M* = 0.623	*M* = 2.545	≤ 0.01
	*SD* = 0.227	*SD* = 0.492	
Speed-congruence-score	*M* = 89.148	*M* = 57.895	≤ 0.01
	*SD* = 5.306	*SD* = 8.580	
Response time during speed-congruency test	*M* = 1.630	*M* = 1.643	0.903
	*SD* = 0.432	*SD* = 0.539	

Of the synesthetes, 77.5% declared to perceive other forms of synesthesia. On average 2.6 ± 2.9 (ranging from 0 to 10) additional forms of synesthesia were reported.

In 14 synesthetes the color perception occurs in the mental image, which according to Grossenbacher corresponds to a classification as “associators.” 26, on the other hand, belong to the “projectors,” in which the synesthetic perception occurs in direct proximity to the grapheme.

### 3.3 BDNF level

**Figure 2:**
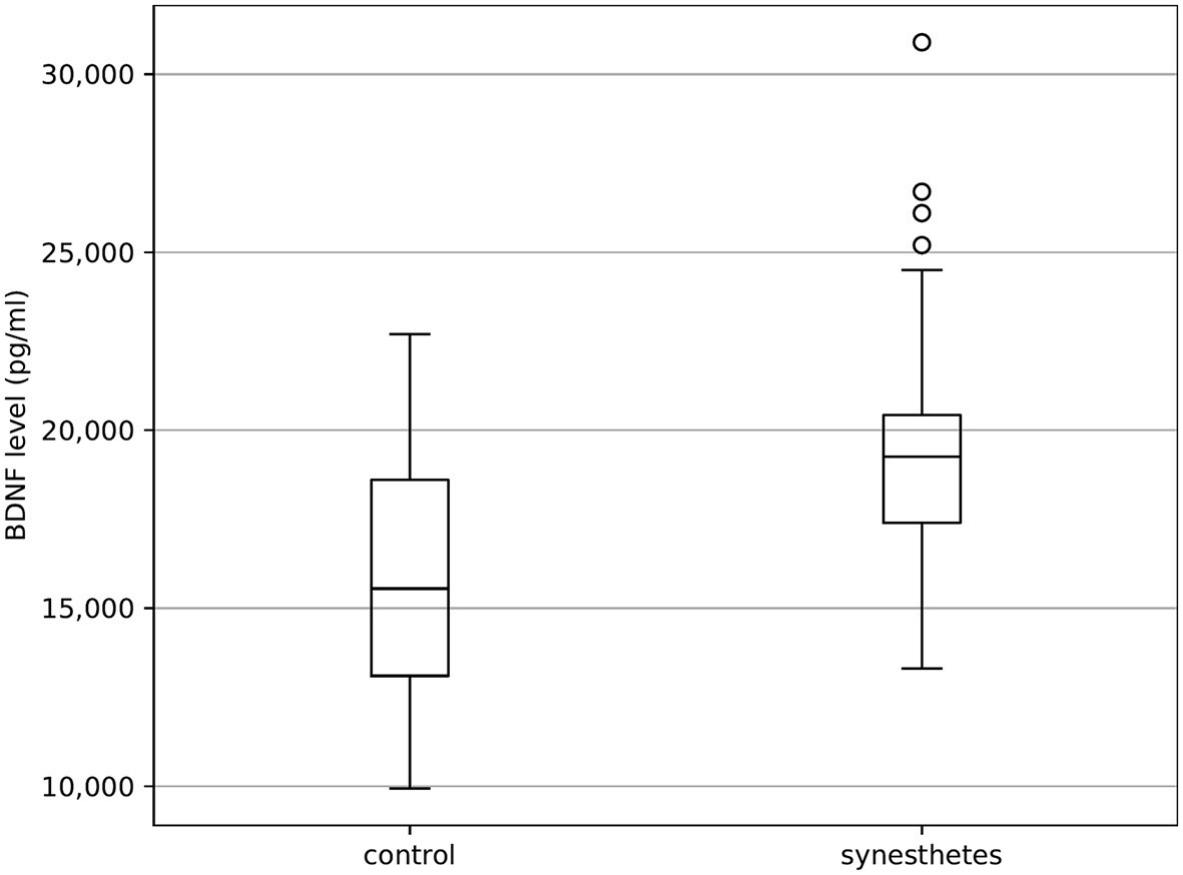
Boxplot of the comparison of BDNF levels between synesthetes and non-synesthetes (control) are plotted as median values in pg/ml. The white boxes contain values between the upper and lower quartile of the data, whereas whiskers are used to indicate minima and maxima of the data (quartile + 1.5 times interquartile range). Values outside this range are considered outliers and marked as white circles.

As shown in Figure 2, analysis revealed that the synesthetes had higher BDNF levels compared to the control group (synesthetes: 19.572.50 ± 3,548.78 pg/ml, controls: 15,837.89 ± 3,299.86 pg/ml; *t*_(76)_ = 4.807, *p* < 0.001).

The study did not find any clinically significant outliers, and thus all the results are included. Further, the outlier-adjusting analysis also reports increased BDNF levels in synesthetes [synesthetes: 18,722.22 ± 2,459.63 pg/ml, controls: 15,837.89 ± 3,299.86 pg/ml; *t*_(72)_ = 4.245, *p* < 0.001].

A difference in BDNF levels between the ELISA kits was not seen [1st ELISA-KIT: 17,858.96 ± 2,256.07 pg/ml, 2nd ELISA-KIT: 17,647.18 ± 5,056.42 pg/ml; *t*_(76)_ = 0.239, *p* = 0.812]. To determine the influence of diurnal cyclic variations on BDNF levels, blood samples were categorized according to the time of blood collection. Here, there was also no effect of time of day [blood collection in the morning (10–12 h): *N* = 30, M: 17,953.33 ± 3,240.02 pg/ml; blood collection at noon (12–15 h): *N* = 29, M = 16,797.93 ± 3,008.32 pg/ml; afternoon blood collection (3–6 pm): *N* = 19, M = 18,894.74 ± 5,555.73 pg/ml; *F*_(2.75)_ = 1.766, *p* = 0.178]. In addition, the storage time of the samples was also examined [synesthetes: 10.3 months ± 4.4, control group: 5.2 months ± 1.3; *t*_(76)_ = 6.759, *p* < 0.001].

Similarly, no correlation was found between the number of other synesthesia forms and serum BDNF levels [ranging from only grapheme-color synesthesia to 10 other synesthesia forms: *r*_(38)_ = 0.006, *p* = 0.968]. Projectors and associators also have comparable BDNF values [projectors: 18,214.29 ± 2,324.74 pg/ml; associators: 20,303.85 ± 3,904.71 pg/ml, *t*_(38)_ = 1.829, *p* = 0.075].

Moreover, serum BDNF level does not correlate with age [*r*_(76)_ = 0,0.32, *p* = 0.784] nor BMI [*r*_(76)_ = −0.125, *p* = 0.277]. No influence of education level on the BDNF concentration could be determined either [university: *N* = 32, M = 17,634.38 ± 3,627.01 pg/ml; high school: *N* = 30, M = 17,698.00 ± 4,612.31 pg/ml; secondary school: *N* = 7, M = 18,093.75 ± 3,040.94 pg/ml; *F*_(2, 75)_ = 0.77, *p* = 0.926].

A final multivariate linear regression model with backward selection was not performed because no influencing factor could be determined to have a univariate significant effect on the outcome variable.

## 4 Discussion

This study aims to answer the question if the BDNF serum concentrations, in their role as a marker of neuroplasticity, can be utilized as proxy to find a connection to the different existing explanatory models for the etiology of synesthesia. At the time of this study, it remains debated whether synesthesia is a relic of early childhood development or if the cross-sensory percepts represent a more highly developed form of consciousness. In other words, it may correspond to a differentiated and further developed state that is associated with increased neuroplasticity. As a first line of inquiry, a statistical link between BDNF serum concentrations and the occurrence of synesthesia was examined.

In this study, synesthetes were shown to have statistically significantly higher BDNF serum levels, hence a higher neuroplasticity, than a comparable control group. There are numerous factors that can influence neuroplasticity and need to be accounted for. However, from the gathered data, no significant differences were observed between the two groups, except for the storage time of the serum samples. Previous studies have demonstrated that only extreme storage times influence BDNF levels, leading to a reduction in its level (Trajkovska et al., 2007; Bus et al., 2011; Polyakova et al., 2017). Thus, these findings are not in conflict with the results of the study, as the synesthetes had increased BDNF levels despite the longer storage time.

One approach proposes that synesthesia may be an archaic phenomenon, left over from brain development in early childhood. This theory suggests that all infants have a synesthesia-like cross-modal connectivity, which regresses over the course of brain development due to experience-dependent neuronal synaptic pruning and/or neuronal inhibition (Maurer and Mondloch, 2005; Maurer et al., 2013; Spector and Maurer, 2009). This “neonatal synesthesia theory,” predicts a lower incidence of synapse formation during adulthood. However, the findings of this investigation suggest that synesthetes have increased BDNF levels. Therefore, it is possible that synesthesia is not only a mere vestige in the studied grapheme-color synesthesia group. Instead, one could assume that synesthesia, in the sense of a hyperconnected brain, is a pre-developed variant of consciousness with particularly pronounced neuroplasticity and high neutrophin levels. Further investigations are necessary to rule out any factors that are influencing BDNF levels.

At a first glance, the results of this study are more in line with research conducted by Simner and Bain, who report further advancement of synesthetic perception in children (Simner and Bain, 2013). The findings indicate that out of eight synesthetes tested in 2009, aged 6/7 or 7/8, five still exhibited synesthetic perceptions at the ages of 10 and 11, respectively. Furthermore, a child who was previously classified as a “high memory non-synesthete” has now been reclassified as part of the synesthesia group. In 2013, the synesthetic perceptions showed a shift toward a system of fixed, constant inducer/concurrent pairings. It is uncertain whether this progress could be associated with an enhancement in neuroplasticity. However, it has also been shown that some traits are lost during neuropsychological development, which the authors described “synesthetic demise.” For example, after a span of 3 years, one child only perceived letters in color, but no longer numbers as previously had been the case. These observations imply that synesthesia may be linked to dynamic processes of neuronal inhibition and pruning, in addition to increased connectivity between different sensory areas during infant brain development. Research on marker neurotrophins, such as BDNF here, provides a window into these phenomena.

As synesthesia involves the connection of two independent sensory modalities through neural pathways to create a unique sensory experience, the increased levels of BDNF in synesthetes could be interpreted in a way that the unusual sensory coupling might be a byproduct of increased neuroplasticity.

Interestingly, BDNF levels are also related to cognitive performance as some signaling pathways activated by BDNF play a crucial role in distinct memory processes, as explained in a review by Bekinschtein et al. (2014). Notably, clinical-phenomenological synesthetes are observed to display a high level of cognitive function. Despite conflicting results in this area, there is also clear evidence of consistencies between synesthesia, creativity, and memory functions. Synesthetes are often associated with elevated levels of creativity (Ward et al., 2008; Rothen and Meier, 2010), mental imagery (Spiller et al., 2015), lucid dreams (Khallieva et al., 2022), and improved ability to recall certain aspects of the memory (Rothen et al., 2012; Lunke and Meier, 2018). These observations are thoroughly discussed in a recent work by Ward (2013). Although synesthesia is not a mechanism for compensating for age-related memory decline, research has found that the memory advantage of synesthesia remains independent of age and is maintained in old age (Mealor et al., 2020). It remains an open question whether synesthetes benefit a priori from the possibility of polyvalent memory content recording. Either synesthetic perception itself might be responsible for higher mental performance, or certain structural changes in the brain, possibly induced by increased neuroplasticity, potentially lead to a memory advantage (Rothen et al., 2012). However, a recent study by Ovalle-Fresa et al. (2021) has shown that visual memory ability is not related to synesthesia itself, but rather to visual perceptual ability. In their study, 27 individuals with grapheme-color synesthesia and 27 without the condition but with comparable visual ability exhibited enhanced memory capability when compared to a random non-synesthetic control group (Ovalle-Fresa et al., 2021).

It has not yet been determined whether the enhanced neuroplasticity shown in this study leads to the development of synesthesia or if synesthesia causes the enhanced neuroplasticity and hence the BDNF serum levels in healthy adult synesthetes. If the latter is the case, it should be noted that elevated BDNF levels are not necessarily specific to the phenomenon of synesthesia. Also, it cannot be ruled out that a third, unknown factor is responsible for the occurrence of both synesthesia and enhanced neuroplasticity. The elevated BDNF levels could be a side effect of increased sensory experiences and neuroplasticity, so that they could also occur in people without synesthesia who generally have a wealth of sensory experiences and are intensively engaged with their environment. The study by Ovalle-Fresa et al. (2021) yielded comparable results in relation to improved memory performance.

Additionally, individuals with synesthesia show distinctive personality traits and differ in their cognitive abilities and emotional processing compared to those without this condition. Rouw and Scholte (2016) found that, compared to non-synesthetes, synesthetes have higher scores in openness and neuroticism, but lower scores in conscientiousness. Furthermore, increased imagination and emotionality were observed, with slightly higher intelligence scores also being found. It appears that an increase in the number of distinct types of synesthesia is associated with a more pronounced cognitive profile. The differences in personality cognitive abilities and perception are consistent with the observations of Chun and Hupé (2016), who emphasize, however, that some of these traits may be direct concomitants of synesthesia itself, while others may have developed indirectly over time through specific experiences, such as a more artistic lifestyle. In their study, Ward and Filiz (2020) suggest that these neurocognitive differences also have a heritable component. They therefore postulate the existence of a “synesthetic disposition” in the general population, which predisposes to the development of synesthesia and manifests itself as a specific cognitive profile.

BDNF has also been linked to a number of personality traits, as evidenced by studies conducted on healthy individuals without mental disorders. Decreased serum BDNF levels have been found to correlate with elevated harm avoidance scores, as demonstrated by Minelli et al. (2011). Notably, this correlation was observed for serum levels but not for BDNF polymorphisms. Yasui-Furukori et al. (2013) reported an association between low BDNF levels and increased self-determination scores. Moreover, neuroticism has been demonstrated to correlate negatively with BDNF (Lang et al., 2004). According to this, synesthetes should have lower BDNF levels, but as this study shows, they are elevated. The interaction of BDNF, personality, and synesthesia appears to be complex and potentially influenced by additional variables not included in this study. To gain a deeper understanding of this relationship between neuronal attributes, altered cerebral function and structure, and the associated altered perception and personality development, further research is required that specifically examines personality traits in relation to BDNF in individuals with synesthesia.

A comparison of the results of healthy subjects with cognitively impaired ones, such as stroke patients and autism spectrum, etc., in other studies, suggests some parallels between neuroplasticity and the development of synesthesia as a side effect of the body's response.

For instance, there is growing evidence in the literature that the brain is able to recover certain brain functions after damage, such as occurs during a stroke, through a combination of neurogenesis, reduced apoptosis and reorganization. Liu et al. (2020) provide a comprehensive review of the role of BDNF and its signaling pathways in this context and discuss recent research using BDNF as a therapeutic agent in stroke rehabilitation (Liu et al., 2020). Indeed, stroke-related changes in neuroplasticity appear to be directly related to BDNF levels. According BDNF levels decrease immediately after stroke, however, additional therapy and exercise can further increase these levels again and positively influence the repair mechanisms (Mojtabavi et al., 2022). Increasing BDNF levels seemingly leads to a 'rewiring' to restore brain function, either by making new neuronal connections, strengthening weak neuronal connections between different brain areas, or by other cortical structures that compensate for the function of the damaged brain region. In this context, it would be interesting to look whether synesthetes show faster recovery and restructuring after brain damage due to their increased markers of neuroplasticity.

In rare cases, acquired synesthesia may occur due to the brain reordering lost neuronal pathways during rehabilitation or by chance. For instance, a 45-year-old man experienced multimodal synesthesia after thalamic lesion, which persisted for 3 years (Fornazzari et al., 2012). Correspondingly, Beauchamp and Ro (2008) observed similar results with a female subject using BOLD-fMRI after a stroke. Brain neuroplasticity led to the development a sound-tactile synesthesia rather than the restoration of the initial neuronal connections (Beauchamp and Ro, 2008). Furthermore, a correlation between synesthesia and increased creativity was observed in a recent case report following traumatic brain injury (Abou-Khalil and Acosta, 2023).

These cases provide a unique insight into the relationship between neuroplasticity and synesthesia, as former lead to development of synesthesia and not the other way around. Given the link between BDNF and neuroplasticity, synesthetes appear to be a healthy alternative for studying the effects of BDNF independent of diseases.

It is unclear whether synesthesia, as a normative variation of human consciousness, could represent a form of awareness that has the potential to prevent developmental disorders or compensate for them in the sense of a cognitive reserve capacity (Zedler and Rehme, 2013). Extrapolating, one could hypothesize that the phenomenon of synesthesia may be associated with enhanced resilience to psychiatric illness. However, to fully investigate this issue, additional research effort is required.

Carmichael et al. (2019) investigated the presence of synesthesia in a large population, while recording the comorbidity with 23 different diseases. Anxiety disorder was the only identified concomitant disease. However, the authors noted that the search for co-occurrence is statistically challenging when both phenomena have low prevalence (Carmichael et al., 2019).

Synesthesia can also be linked with autism spectrum disorder (ASD). Neufeld et al. (2013) found the 467 prevalence of synesthesia in individuals with Asperger syndrome to be 17.2%. Both phenomena are associated with atypical brain connectivity and sensory activation, which may be due to altered synaptic neuroplasticity.

The etiology and pathology of ASD, and its various individual manifestations, are intricate and heterogeneous. The mechanisms behind this neurodevelopmental disorder remain largely unknown. However, it is hypothesized that an impaired structural and functional neuroplasticity results in the disruptions of neuronal circuits, ultimately leading to changes in the brain's perception and information processing. In their review, Zong et al. investigate these molecular biological mechanisms underlying “synaptic dysfunction/disturbance” in detail, pointing out that the literature currently reports both an increase and a decrease in the so-called “synapse/spine density” (Zong et al., 2023). The reduction is caused by several factors, including dysregulation of the BDNF/TrkB signaling pathway. On the other hand, an enlarged brain volume during the initial developmental phase, resulting from a greater proliferation and increased quantity of neurons (Courchesne et al., 2011), has also been observed and is therefore also linked to BDNF. This has also been reported in cases with increased neural density, there is a deficiency in synaptic elimination (Tang et al., 2014). This is similar to Maurer's “neonatal pruning hypothesis” for synesthesia, which suggests that synesthesia is a relic of early development (Maurer and Mondloch, 2005). Although establishing a causal relationship between ASP and synesthesia in such instances is challenging, statistical evidence establishes an association between them (Carmichael et al., 2019). However, whereas the prevalence of synesthetes in individuals with ASD is increased, it remains unproven whether the frequency of ASD is elevated in those with any type of synesthesia.

It has been found that the co-occurrence of synesthesia and autism in an individual may contribute to the emergence of the same savant skills (Hughes et al., 2017). The recent case report by Riedel et al. highlights that this is related to increased brain connectivity. Although individuals with ASD often experience lifelong difficulties with social communication and interaction, as well as limited, repetitive and restrictive behaviors, interests, and activities, regardless of their intelligence level, the synesthete LP appears to have the ability to acquire languages, like Spanish, using his synesthetic perception (Riedel et al., 2020). Not only being relic of early childhood, Synesthesia, in such cases, appears more like a form of cognitive (further) development. This could be due to an elevated BDNF level and significant neuroplasticity.

BDNF levels in individuals with ASD compared to the norm are controversially reported in the literature, with reports suggesting increased BDNF, although often with some heterogeneity (and Barbosa et al., 2020, for current meta-analysis; refer to Liu et al., 2021). The meta-analysis by Saghazadeh and Rezaei (2017) showed elevated BDNF levels in blood, but not in serum, plasma or cord blood, whereas Zheng et al. (2016) demonstrated elevated BDNF levels in children, but not in adults. In contrast, Hashimoto et al. (2006) reported BDNF decrease in males with ASD.

It has been shown that there is a link between synesthesia and ASD and that both conditions are characterized by altered BDNF levels. BDNF is believed to be a potential mediating factor, with a positive impact on cognitive abilities and the brain's ability to adapt to illness.

In line with this, the literature reports increased levels of BDNF associated with physical activity, resulting in improved memory and learning (Tang et al., 2008; Huang et al., 2014). This observation is consistent with recent meta-analyses which showed that physical activity also benefited people with ASD (Ferreira et al., 2019; Huang et al., 2020) or depressive disorders (da Cunha et al., 2023). According to the neurotrophin hypothesis, a model that explains depression, changes in neuronal plasticity and altered BDNF levels are crucial (Jaggar et al., 2019 for more detailed information; see Yang et al., 2020). This degenerative disease is associated with lower serum BDNF levels (Karege et al., 2002a), which can be elevated to a basal level through antidepressant therapy (Shimizu et al., 2003), as has been established by recent meta-analyses (Zhou et al., 2017; Shi et al., 2020).

According to the results of this study, the neuroplasticity of synesthetes is increased even without physical activity or therapy. However, it has not yet been investigated how a disease with reduced synaptogenesis would behave in the presence of synesthesia simultaneously. If synesthesia indeed results in higher BDNF levels in such cases, it is worth exploring whether synesthesia can maintain brain function via alternative neuronal networks and potentially delay or even reverse degeneration in such a scenario. Such a result would highlight the pronounced neuroplasticity, reflecting a hyperconnected brain and suggested to result from or be associated with synesthesia.

If synesthesia indeed results in higher BDNF levels in such cases, it could be worthwhile to explore whether synesthesia is helping to maintain brain function via alternative neuronal networks and potentially delay or even reverse degeneration in such a scenario or if the pathogenesis is unaffected.

However, the fact that an increased familial incidence has been identified argues against a compensatory mechanism theory. Since individual differences in the type and perception of synesthesia have been described within the same family, synesthesia seems to be based on a genetic predisposition with an environmental influence (Barnett et al., 2008). A genetic (Ward and Simner, 2020) for synesthesia has not yet been found, only a locus heterogeneity (Asher et al., 2009; Tomson et al., 2011; Ward and Simner, 2020) has been described, which interestingly is associated to axogenesis (Tilot et al., 2018) and therefore neuroplasticity, as already described above.

Nevertheless, it cannot be excluded that synesthesia is an evolutionary coincidence or a windfall.

### 4.1 Limitation

There are a multitude of considerations when comparing the study presented here with others. From a methodological standpoint, the measurement of BDNF is a subject of controversy, as different measurement procedures (including Elisa kits, serum vs. whole blood vs. plasma, total BDNF vs. mature BDNF or proBDNF, for further details see Elfving et al. (2010b); Polacchini et al. (2015); Gejl et al. (2019), in addition to measurement conditions (such as temperature, anticoagulant, centrifugation time, and clotting time, for further information see Maffioletti et al. (2014); Tsuchimine et al. (2014); Amadio et al. (2017); Gejl et al. (2019) and storage conditions (including duration and temperature, for more details see Trajkovska et al., 2007; Bus et al., 2011; Polyakova et al., 2017), may significantly impact the recorded concentrations. It is beyond the scope of this report to provide a detailed discussion of the various methods employed. It was ensured that all samples were treated equally to avoid any data bias.

Only healthy participants were included in this study. However, their overall health status is relevant to consider when comparing with other studies. It is important to note that certain diseases, for example metabolic syndromes, not covered in this research, have a direct impact on BDNF levels (for more details see Motamedi et al., 2017). BDNF concentrations can also be influenced by general lifestyle factors such as diet, physical activity, tobacco and alcohol consumption, exposure to stress, and demographic characteristics such as age and gender (Lommatzsch et al., 2005; Bus et al., 2011; Huang et al., 2014; Singh et al., 2014).

The study demonstrated a difference in BDNF levels between individuals who experience synesthesia and those who do not. Future studies are needed to determine if the observed differences in markers of neuroplasticity are linked to the neuronal mechanisms of synesthesia, hence measures of neural connectivity and/or perceptual function in the synesthetes. This would help to gain a deeper understanding of the impact of the observed BDNF differences in the context of synesthesia.

## 5 Conclusion

In this study, the connection of serum BDNF concentrations in healthy subjects with and without grapheme-color synesthesia was investigated, as a route to the neurobiological underpinnings of synesthesia, which remains poorly understood. A statistically significant elevation in serum concentration was observed in grapheme-color synesthetes suggesting that synesthetes exhibit enhanced neuroplasticity. This could indicate that the anatomy and function of the brain in synesthetes was altered as a function of use (in parts due to character traits and cognitive styles), resulting in more differentiated (further) development. It remains to be clarified whether the increase in BDNF levels observed in this study is caused by the development of synesthesia or is a consequence of it. From the gathered data, no cross correlations between enhanced BDNF levels and other data than the presents of synesthesia could be found.

## Data Availability

The raw data supporting the conclusions of this article will be made available by the authors, without undue reservation.

## Footnotes

^1^The Synesthesia Battery (2023). Available at: https://synesthete.ircn.jp/home (accessed November 21, 2023).
